# Willingness to Participate in HIV Vaccine Trials among Men Who Have Sex with Men in Chennai and Mumbai, India: A Social Ecological Approach

**DOI:** 10.1371/journal.pone.0051080

**Published:** 2012-12-04

**Authors:** Venkatesan Chakrapani, Peter A. Newman, Neeti Singhal, Jhalak Jerajani, Murali Shunmugam

**Affiliations:** 1 Centre for Sexuality and Health Research and Policy, Chennai, India; 2 The Humsafar Trust, Mumbai, India; 3 Factor-Inwentash Faculty of Social Work, University of Toronto, Toronto, Ontario, Canada; University of Ottawa, Canada

## Abstract

**Background:**

Recruitment of low- and middle-income country volunteers from most-at-risk populations in HIV vaccine trials is essential to vaccine development. In India, men who have sex with men (MSM) are at disproportionately high risk for HIV infection and an important population for trial recruitment. Investigations of willingness to participate (WTP) in HIV vaccine trials have focused predominantly on individual-level determinants. We explored multi-level factors associated with WTP among MSM in India.

**Methods:**

We conducted 12 focus groups (n = 68) with low socioeconomic MSM in Chennai and Mumbai, and 14 key informant interviews with MSM community leaders and service providers. Focus groups/interviews were recorded, transcribed and translated into English. Two bilingual investigators conducted thematic analysis using line-by-line coding and a constant comparative method, with member-checking by community representatives.

**Results:**

Factors associated with WTP were evidenced across the social ecology of MSM–social-structural: poverty, HIV-, sexual- and gender non-conformity stigma, institutionalized discrimination and government sponsorship of trials; community-level: endorsement by MSM community leaders and organizations, and fear of within-group discrimination; interpersonal: anticipated family discord, partner rejection, having financially-dependent family members and disclosure of same-sex sexuality; and individual-level: HIV vaccine trial knowledge and misconceptions, safety concerns, altruism and preventive misconception.

**Conclusion:**

Pervasive familial, community and social-structural factors characteristic of the Indian sociocultural context may complicate individual-focused approaches to WTP and thereby constrain the effectiveness of interventions to support recruitment and retention in HIV vaccine trials. Interventions to reduce stigma and discrimination against MSM and people living with HIV, capacity-building of MSM community organizations and transparent communications tailored to the knowledge and educational level of local communities may support meaningful engagement of MSM in HIV vaccine trials. Vigilance in providing fair but not excessive compensation and healthcare benefits and in mitigating preventive misconception are warranted to support ethical conduct of trials among MSM in India.

## Introduction

India, with a population estimated at 1.2 billion, 2.4 million people living with HIV and 120,000 new HIV infections every year [1], has the highest disease burden of HIV in Asia. Overall HIV prevalence remains low, but is estimated at 7.4% among men who have sex with men (MSM) [2], more than 20 times the national average (0.3%) [1].

Efforts to develop an effective HIV vaccine are proceeding internationally. Yet out of over 235 trials thus far implemented, including more than 25,000 volunteers, only two phase I trials have been completed in India [3]. The International AIDS Vaccine Initiative (IAVI), recognizing the importance of India to HIV vaccine development, undertook consultations with multiple stakeholders: government representatives, scientists, nongovernmental organizations (NGOs) and community-based organizations (CBOs), including MSM in Chennai [4]. Such preparedness efforts, including stakeholder engagement and assessment of willingness to participate (WTP) among populations with high HIV incidence, are crucial to the successful implementation of HIV vaccine trials [5], [6].

Over 60 published studies of WTP in HIV vaccine trials have been conducted in high-, middle- and low-income countries [7]–[9]. Among these, one study of four implemented in India [10]–13 included a small subsample of MSM, but no discussion of population-specific concerns [11]. To support the safe and ethical implementation of HIV prevention trials among MSM in India, we explored factors associated with WTP among MSM in two large Indian cities, feasible locales for cost-effective HIV prevention trials.

## Methods

### Ethics Statement

The study protocol was approved by the research ethics boards of the University of Toronto and The Humsafar Trust. All participants provided written informed consent to participate in this study.

### Participants and Recruitment

We adopted a community-based collaborative research approach [14] by engaging community-based organizations (CBOs) working with MSM in Chennai (Social Welfare Association for Men and Sahodaran) and Mumbai (The Humsafar Trust) throughout all stages of study design, instrument development, data collection and interpretation.

We conducted focus groups with MSM service users of local CBOs and MSM peer outreach educators. In Chennai, outreach educators had been part of IAVI-sponsored community consultation meetings. We conducted key informant (KI) interviews with MSM community leaders, peer counselors and healthcare providers. Focus group participants were recruited by word of mouth by trained peer outreach staff, a method we have used successfully in order to mitigate stigma associated with written recruitment materials and to be broadly inclusive of low socioeconomic MSM, some of whom are illiterate [15].

Eligibility criteria for focus group participants were self-identification as MSM, aged 18 years or above and being able to provide informed consent. We used stratified purposive sampling to include perspectives of different MSM subgroups. We conducted separate focus groups for kothis (feminine gender expression and generally receptive partners in anal sex), panthis (masculine gender expression and generally insertive partners in anal sex) and double-deckers (adopt both insertive and receptive roles). We used purposive sampling to select ‘information-rich’ [16] key informants.

### Data Collection

We used a semi-structured interview guide to explore factors that might influence WTP in a future HIV vaccine trial. Focus groups (60–90 minutes) and KI interviews (45–60 minutes) were conducted in participants’ native language (Tamil in Chennai; Marathi or Hindi in Mumbai); a few KIs chose to be interviewed in English. Focus groups and interviews were audio-taped, transcribed verbatim, redacted and translated into English. We checked the accuracy of transcripts by randomly selecting 20% and comparing them with the respective audio files.

### Data Analysis

Focus group and interview data were explored using narrative thematic analysis [17] and a constant comparative method [18]. We developed a code book based on the interview guide and available literature and added codes/categories that emerged during analysis. Two bilingual investigators individually analyzed each transcript, followed by team analysis using NVivo 7. Differences in coding were resolved by consensus in research team meetings. We discussed findings and interpretations in meetings with field research teams and community representatives in each site, with their input included as ‘feedback data,’ a form of member checking to increase the validity of the findings [19].

## Results

### Sociodemographic Characteristics

From June 2010 to June 2011 we conducted seven focus groups (n = 43) in Chennai and five (n = 25) in Mumbai (mean age = 27.8 years). Sociodemographic characteristics of focus group participants and KIs are listed in Table 1. The majority (52%) of focus group participants identified as kothi, one-fifth (21%) as double-deckers and about one-fifth (18%) panthis. Sixteen-percent (n = 11) were married. Most participants were of lower socioeconomic status, characteristic of the clientele of our CBO partners [15], [20], [21]: 25% had less than high school-degree education and half completed only high school. Over two-thirds were employed in low wage jobs (44%), unemployed (15%) or sex workers (12%).

**Table 1:** Sociodemographic characteristics of study participants (n = 82).

Characteristics		Focus group participants (n = 68)	Key informants (n = 14)
Age (years)	Mean	28	40
	Range	20–46	29–60
Marital status	Unmarried	57 (84%)	11 (79%)
	Married	11 (16%)	3 (21%)
Education	Primary (5th grade or less)	10 (15%)	
	6th grade to 11th grade	22 (32%)	
	High school degree	18 (26%)	
	College degree	18 (26%)	14 (100%)
Employment	CBO staff	15 (22%)	8 (57%)
	Daily-wage laborer	16 (24%)	
	Private company staff	19 (28%)	
	Sex work	8 (12%)	
	Unemployed	10 (15%)	
	Head of CBO/professional association		4 (29%)
	Medical doctor		2 (14%)
Sexual identity	Kothi	35 (52%)	
	Double-decker	14 (21%)	
	Panthi	12 (18%)	
	Versatile/bisexual	4 (6%)	
	Other	3 (4%)	

Note: CBO = community-based organization.

We conducted nine KI interviews in Chennai and five in Mumbai (mean age = 39.5 years). KIs included experienced staff (project managers and outreach coordinators) and directors of MSM CBOs, and three medical and social service professionals.

### Stigma and Discrimination

#### Sexual prejudice and HIV stigma

Multiple dimensions of stigma, based on sexuality, gender non-conformity and HIV-status, were pervasive barriers to WTP: “If you go in for a vaccine trial and want to be a part of it you are admitting to be at high risk … you are admitting to be one of the marginalized populations” (KI1, Chennai). A focus group participant explained:

People seeing us from outside will not know about us. They may not know we are [MSM]. But they might think that these people [trial volunteers] are at high risk and that is why they participate. They may look down upon us…look at us in a different manner. (Kothi, FG2, Mumbai).

Some KIs implicated HIV vaccine preparedness efforts targeting MSM as themselves stigmatizing: “But why with MSM? That means directly you are stigmatizing us–that he is MSM, lots of sex–and that is why you are doing this with the MSM population” (KI2, Chennai).

Participants expressed concerns about discrimination–in their community and from trial staff. A kothi informant reported, “In our community, even if a person is thin he would be suspected of HIV and discriminated. So what if others come to know that I get registered in a trial?” (KI4, Chennai). A panthi expressed concern about discrimination from medical providers:

Doctor’s [in trial] behavior is main, if he talks respectfully with the [MSM CBO] staff and [trial volunteers] then it will obviously make a difference. If doctor’s behavior is good then we will take part in the study. The main thing is how you talk. (Panthi, FG5, Mumbai).

#### Confidentiality and fear of being “outed”

Participants who expressed intentions to volunteer for a trial wanted assurances of privacy and confidentiality, including acceptable locales and hours of operation: “It would be fair if complete confidentiality of information about the participants is maintained. There must be privacy in the place where the trial is conducted and no one else should know who participates” (Kothi, FG7, Chennai). However, participants expressed concerns about the level of confidentiality in HIV prevention trials:

One fear is fear of loss of respect. …I may be in [MSM] community but I am in the closet. What if I go [to trial], the doctor will come to know and he blabs it out to someone? One will tell ten, ten will tell twenty, then…? (Kothi, FG1, Chennai).

Alternately, a kothi explained: “Those who are living openly… if they get to know the benefits of study then they will participate without any fear” (Kothi, FG2, Mumbai).

Panthi and double-decker participants, who are seen by kothis as not open about their sexuality and as generally remaining hidden, also expressed interest in participation; some reported they were not concerned about disclosure of their sexuality, believing the risk of being “outed” is less likely because they are masculine-looking and unlikely to be suspected of being ‘homosexuals’.

#### Vaccine-induced seropositivity (VISP)

During focus groups and interviews, the interviewer/facilitator explained that some trial vaccines may induce HIV-seropositivity as a normal immune response. Some participants revealed difficulties in comprehending the difference between VISP and actual infection. An MSM community leader (KI1) from Chennai reported that if the possibility of VISP is explained to potential volunteers, “they will get real scared and confused, and run away.” The major concern, however, even among those who understood that VISP does not mean HIV infection, was due to HIV stigma and challenges in convincing others that one is not HIV-positive. A further concern was barriers to overseas employment due to HIV testing requirements.

### Endorsements from Government, CBOs and Peers

Participants generally reported that if MSM CBOs endorse and support a vaccine trial, many MSM would volunteer. Government sponsorship of trials and endorsement by peers and past participants were also valued sources of trust. Trust in CBOs, particularly among kothi-identified MSM who were the major service users, was reported to the extent that they would not even ask questions before agreeing to participate.

If the [CBO] project manager calls us, all of us will participate. We will participate in the study purely for them. We will come for the organization; if they tell us to go, then we will go. (Kothi, FG1, Chennai).

Some participants suggested that recruitment of potential trial volunteers be conducted only through MSM CBOs, and that these are more reliable than the government: “The government is here today, gone tomorrow; government can change anytime. But if [CBO name] is with us…then we will do it” (Kothi, FG4, Mumbai).

Importantly, MSM’s unbridled trust in CBOs was described as double-edged by community leaders who manage CBOs. A community leader explained:

We have established rapport with the community over a period of several years. What if something happens in the trial? Who will they blame? They will blame us! They will say, ‘You should have warned us. We trusted you.’ (KI2, Chennai).

Participants also expressed a desire to meet with former HIV vaccine trial participants to be assured about safety:

You [interviewer] stated that the vaccine was tested among 50 individuals of general population, and it was a successful one…then why don’t you arrange a meeting for us with them? Let them say that, ‘We also volunteered like you. We did not have any problem.’ Like that, if they give us 100-percent confidence, they [MSM] will come definitely. Because they have already gone through all levels, they know everything. (Kothi, FG1, Chennai).

Some participants expressed confidence in any government sponsored vaccine trial, reasoning that the government would not make decisions that harm people. A participant referred to a recent government crackdown on sales of expired medications: “These days government has awakened and located all drugs that were expired; so in such a situation…if bravely it [trial] is implemented through organizations like [CBO name], we will very well welcome the trial” (Kothi, FG2 Chennai).

However, a few participants noted that their trust in government was contingent on transparency: “All information about vaccine trials including previous experiences should be shared with us and if awareness is created by the government *then* we will trust the government” (Kothi, FG1, Chennai).

### Financial Concerns

Monetary compensation for trial participants was seen as a “must.” Community leaders indicated that participants should be compensated for their time and travel expenses and warned against taking the community “for granted.” Participants described monetary compensation as a “duty” of trialists, something to which volunteers are entitled. Participants stressed the need for monetary compensation for kothis engaged in sex work, who live in poverty: “If kothis in *dhandha* [sex work] need to participate, then some money other than travel allowance has to be given, since their earnings depend on their sex work” (KI1, Chennai).

MSM community leaders, however, expressed concerns about excessive compensation that would be tantamount to coercion: “Some MSM do not trust [trialists] by thinking that they might ‘misuse’ them for money” (KI2, Chennai). Participants also reported that money would be a strong incentive, particularly among MSM in sex work: “Money is the main factor. None would be ready to volunteer for the sake of community” (Kothi, FG5, Chennai); “Kothis in difficult situations will come to participate only if money is shown” (Kothi, FG2, Mumbai). A community leader explained:

There are some MSM who are in the ‘commercial field’ [sex work]. Only they would come forward. If we announce 1000 [Indian Rupees] for the participants of the vaccine [trial], some people are readily available. There are many people in [Chennai] with such issues. (KI2, Chennai).

In contrast, a CBO staffperson characterized the role of monetary compensation as artificially induced by non-governmental organizations (NGOs) outside of MSM communities:

Many would be willing to do it for their community. Kothis going to sex work will participate voluntarily. We have to only blame the NGOs for initiating this custom of [monetary] compensation. (Kothi, FG7, Chennai).

Although some suggested that kothis will participate for the sake of money even if they do not fully understand vaccines and trials, others challenged that notion: “Those who live in poverty will use the opportunity, that too only if they are assured that there are no negative aspects in the study…if they find any negative aspects, even they will refuse to come” (Kothi, FG6, Chennai). KIs similarly reported that if VISP is discussed many sex workers would not want to participate as this would be a “barrier to their livelihood” (KI8, Chennai). “[MSM] who engage in sex work may think, ‘Why should we get involved and take unnecessary risk? We shall earn and live in peace till we can’” (KI3, Chennai).

### Concerns about Families and Partners

The variety of permutations of familial and partner concerns demonstrates the pervasiveness of relational factors in WTP. MSM described that having dependent family members was a barrier to WTP due to concerns about who would take care of their parents if they are injured in a trial:

Many kothis have dependent parents to take care. More than any other person, kothis love their parents very much. Hence, I do not know whether they will participate in this vaccine trial. (Kothi, FG6, Chennai).

Some reasoned that it would be easier for MSM who are cut off from their families to participate: “Some *patchai* [‘obviously feminine’] kothis have got out of their parents’ home and live separately. There will not be any problems for them to participate” (KI2, Chennai).

Another stated barrier to WTP among MSM who live with their parents or wife was disclosure of their sexuality: “Even though high court judgment [decriminalizing consensual same-sex relationships] has been announced, MSM cannot tell their parents he is having sex with other guys. Since MSM are hidden in this society, I don’t know how MSM will accept to participate in this trial since that might reveal their sexuality to others” (Kothi, FG5, Chennai). A married man reported that it would not be a problem for him to participate because everyone knows about his sexuality already, nevertheless indicating the presumption about disclosure of one’s sexuality as a function of HIV vaccine trial participation.

Some participants indicated further barriers to WTP among married MSM: “Married [MSM] have sex with their wives; some might not want to [participate] as they would be afraid something might happen. What if virus or something jumps from him to her?” (Kothi, FG6, Chennai). In contrast, a married kothi said that he would participate because he wanted to prevent his wife from getting infected, as condom use with his male partners was not always possible. This also belies preventive misconception as a motivator of WTP.

Some MSM who were financially dependent or had strong emotional connections with their parents expressed concerns about volunteering: “As for me I am a DD [double-decker], I depend more on my family, so I cannot come or decide for any such aspects. But kothis will come in large numbers” (Double-decker, FG3, Chennai).

MSM who were living with parents reported that it was important to tell family members about the trial and get their approval although one need not reveal one’s sexuality to them. They reasoned that if they develop medical complications as a result of participating in the trial their family members would then learn about it after the fact, which might result in family discord:

I don’t think parents will give permission. If I go on my own and if my vaccine works, then my life is made; but if it is not so my life can become bad. So if tomorrow my family comes to know [about participation] they will look at me in a wrong way. (Kothi, FG2, Mumbai).

This again suggests preventive misconception as a motivator of WTP.

Participants explained that family members should be told about trial participation so that families could be compensated in the event of any injury to trial volunteers. Some were hopeful that they could convince their parents to allow them to participate:

I am major (legal]) hence I am participating in this discussion. So, I think it can be clearly shared with our family. At first we will have to understand about it by having discussion with doctor and then we can share it with mother to get her written consent. It is because she is the person who has crossed our age, so we can always get her cooperation too. There is nothing wrong in it. We need to make her understand that there will be many who will get benefited by our participation. (Panthi, FG4, Chennai).

We need to tell our family about it. We need to tell them such a research trial is taking place. They will only appreciate us for taking part in it, they will never discourage us. (Double-decker, FG3, Chennai).

However, some participants questioned the need to inform and get approval from parents: “When I first had sex…at that time I had not asked anyone, so why should I ask now? It is for my safety, I can directly go over there [to the trial]” (Double-decker, FG1, Mumbai).

MSM cohabitating with male partners explored the possibility of sensitizing their partners to gain support for participation or even motivating partners to participate too: “We are sharing both joys and sorrows with our life partners; so, if we explain to him about the vaccine trial then he might support us to participate” (Kothi, FG7, Chennai). But some MSM thought that most male regular partners would be against participation:

If there is a regular partner then he will say, ‘What is the need for you to do this? Aren’t there other people? Only you are there or what? Let others go – you should not. (Double-decker, FG1, Mumbai).

### Knowledge and Misconceptions about HIV Vaccine Trials

Participants demonstrated basic knowledge about vaccines and their role in immunity. When asked what a vaccine is, they used terms like ‘prevention from diseases’, ‘to prevent infection’ and ‘to strengthen our immune system’.

There are a lot of life-taking illnesses like HIV. The vaccine goes inside and gives energy to fight against diseases…it increases that power. The vaccine will do that. This HIV vaccine, it will prepare us more to fight against getting HIV. (Kothi, FG2, Mumbai).

Awareness and understanding of HIV vaccine trials varied across participants. KI community leaders had heard about HIV vaccines, but most community MSM had not. Nevertheless, most participants, including community leaders, MSM CBO staff and community MSM, expressed difficulty in understanding trial-related concepts such as candidate vaccine, placebo-controlled and double-blind trial. Even MSM in Chennai who had attended previous IAVI consultation meetings demonstrated significant misconceptions. Some thought that MSM should not engage in sex when they are enrolled in a trial; some were not sure about the need to use condoms because they thought otherwise trialists could not assess the efficacy of the vaccine; and some feared “that through the HIV vaccine they might get HIV” (Kothi, FG1, Chennai).

KIs and CBO staff shared concerns about possible increases in unprotected sex due to preventive misconception among MSM enrolled in a trial. As a community leader reported: “Understanding of vaccination among the general masses basically translates into that if I have been vaccinated I won’t be infected by that disease. That is why MSM will believe if I have taken vaccine then I am safe from HIV” (KI2, Mumbai). An MSM CBO staff person explained: “MSM will think, ‘I won’t get [HIV] since I have been given vaccine’. So they will start engaging [in sex work] without condoms” (Kothi, FG7, Chennai).

Apart from community leaders, particularly those who had attended IAVI consultation meetings, information on HIV vaccine trials had not reached MSM at the grassroots level. A KI who had previously worked as a field researcher for a formative HIV vaccine study said that he was bombarded by questions, many of which he was unable to answer:

After explaining to MSM [about HIV vaccine trials], they asked me several questions: ‘What is a vaccine? Where this vaccine trial will be done? What benefit will I get if I participate? If I get any problem during participation, will they solve it? How many times will they call us for this vaccine trial? Will they give us travel money? Will that be sufficient for me?’ (KI5, Chennai).

Participants reported difficulties in understanding and accepting HIV vaccine trial concepts, including why a placebo would be used and what a double-blinded study is: “How can you give distilled water [the interviewer mentioned distilled water as an example of a placebo] to people who participate in a trial hoping that they will get the vaccine? Everyone should be given vaccine” (Kothi, FG2, Mumbai). Many focus group participants felt that it would be very difficult for MSM at the grassroots level to understand these concepts because they themselves, some of whom are CBO staff or peer educators, could not “fully understand what all these mean.”

### Safety Concerns and Side Effects

Fear of side effects from candidate vaccines was a key barrier to WTP. A KI noted, “If a vaccine trial is happening…the first things people want to know is side effects; what would be the side effects?” (KI2, Mumbai). Some focus group participants expressed WTP only with assurances of no side effects:

So if I am volunteering I also require some safety. Isn’t it? Are there any chances for side-effects such as allergy? If I am assured that there would be no side effects then there is no issue for me to volunteer. (KI5, Chennai).

A few participants in Mumbai worried about becoming impotent or insane due to an experimental vaccine.

Some participants expressed concerns about vaccine-induced infection: “You said dead virus is put in. How do we know? …After going in it drinks blood and becomes alive, then…? (Kothi, FG6, Chennai).

In the absence of complete assurances against all adverse effects, participants expressed the need for provision of permanent employment, health and life insurance, and paid treatment if one becomes HIV infected or experiences serious side effects in a trial.

If the vaccine fails, if after taking the vaccine I become ‘positive,’ then what about me after that? If the company [trialists] is giving me some policy…some budget for me… either they give money or they give a job that remains a lifetime…then we can take part in that study. (Panthi, FG5, Mumbai).

Participants who were living with their parents or married expected compensation for family members: “I am living with my parents, they are in need of my support, and if I am volunteering in the trial and something happens to me what would be given to my family? (Kothi, FG3, Chennai).

### Altruism

Altruism was expressed as a motivator of WTP to benefit one’s community and the nation: “I am brave and ready to volunteer for the study in order to prevent people like me from infection. I take this stance like I am deputing myself to the military” (Kothi, FG1, Chennai); “I will participate in the trial. It is okay if the vaccine has no effect on me or something not so good happens to me; at least I would have done some good work like how there are some patriots who become martyrs for their country” (Kothi, FG1, Mumbai).

However, a KI cautioned that high levels of WTP among kothis was due to fatalism: “There are more chances for kothis to participate as they often get depressed and come to the situation that they do not want to live anymore. For them participation in a vaccine trial is not a big deal” (KI4, Chennai).

Evidence of fatalism also emerged in focus groups:

“Some [MSM] will have a wish ‘Why not try the [candidate] vaccine on me? Anyway at some point I’m going to get [HIV]. So why not try? If it works then at least I’ll be saved!’” (Double-decker, FG1, Mumbai).

Kothis further expressed that participation in trials might help to combat stigma: “We should definitely participate; not just for us, but also for the general public. They will appreciate us when they come to know that we [MSM] participated in the trials and that was why a vaccine is available now” (Kothi, FG1, Chennai).

## Discussion

In this qualitative investigation among diverse subpopulations of MSM in two large Indian cities, decision-making about WTP was embedded in social-structural, community and familial spheres of influence. By focusing on WTP among MSM in India at the individual level we risk not only misunderstanding and misconceptualizing barriers and motivators to WTP; but we may fail to mitigate ethical challenges as well as circumscribe the impact of interventions designed to support recruitment and retention in biomedical HIV prevention trials.

The preponderance of studies of WTP tend to consider the individual as the foundation for decision-making [7], [9], perhaps reflecting the influence of Western psychology and the dominance of individualism. The crucial role of the global south–with the greatest burden of HIV and availability of cohorts with HIV incidence to power cost-effective HIV prevention trials–suggests the value of adopting a social ecological approach in WTP research and formative HIV vaccine preparedness interventions. For one, conceptualizations of HIV, vaccines and clinical trials are rooted in the sociocultural context [22]. Secondly, as in the present study, although important individual-level concerns are evident, they are embedded in broader familial, community and structural domains.

**Figure 1:**
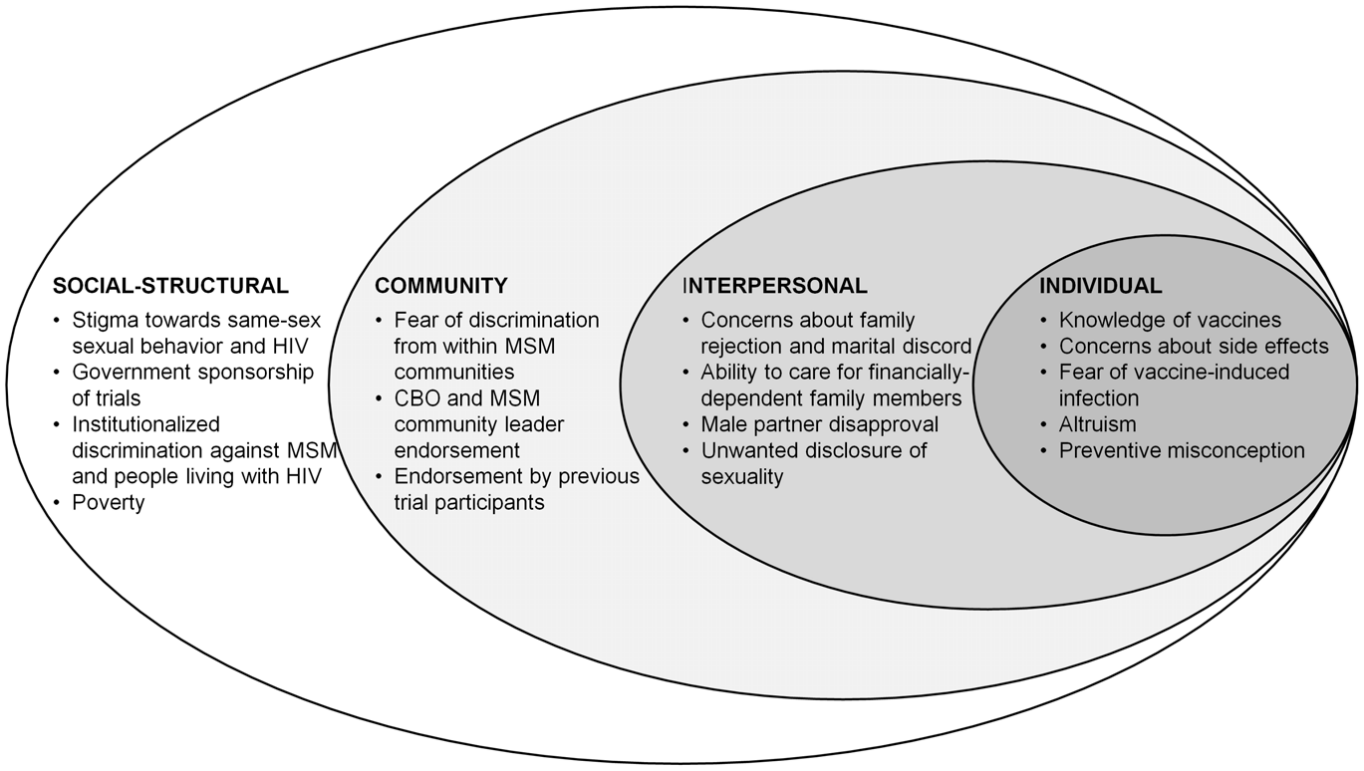
A social ecological model of willingness to participate in HIV vaccine trials among MSM in Chennai and Mumbai, India (n = 82). Note: CBO = community-based organization MSM = men who have sex with men.

Figure 1 is a conceptual model of emergent themes in WTP, organized at social-structural, community, interpersonal and individual levels. As indicated in Table 2, apparently individual-level decisions to participate or not are influenced by social stigma and institutionalized discrimination against same-sex sexuality, gender nonconformity and people living with HIV, and government endorsement of clinical trials (social-structural); endorsements by MSM community leaders and CBOs, peers and past trial participants (community level); and extensive considerations about familial relationships–with parents, male partners and female spouses (interpersonal level). Many of these factors are manifested at multiple levels of the individual’s social ecology.

**Table 2:** Multi-level factors associated with willingness to participate in HIV vaccine trials manifested across the social ecology of MSM in India (n = 82).

Theme	Social-Structural	Community	Interpersonal	Individual
**Stigma & discrimination**	Institutionalized discrimination against MSM & peopleliving with HIV	Fear of discrimination frompeers & local community ifperceived to be HIV+ orat high risk	Family rejection, marital discord & male partner disapproval	Lack of access to competent health care, support services & education
	Stigma & sexual prejudice	Sensitivity & devaluing of characteristics perceived to reinforce stereotypes	Fear of being “outed” as MSM	Shame, low self-worth & fatalism
**Endorsements from trusted sources**	Government sponsorshipof trials	Endorsement by CBOs, community leaders & formertrial participants	Need for permission from parents, wife and/or male partner for participation	Possible overreliance on CBOs challenge individual informed consent
**Financial concerns**	Poverty & under-employment	Loss of income if injuredin trial; loss of income for sexworkers if perceivedto be HIV+	Ability to care forfinancially-dependentfamily members; beingcut-off if dependent	Perceived threat to daily income due to injury or VISP
	Employment discrimination	Difficulties for more effeminate or out MSM in finding work outside community	Ensured employmentif lose job; lifeinsurance	
**Concerns about families & partners**		Confidentiality may bebreached on communitylevel	Fear of marital & family discord if confidentiality in trial is breached	Fear of rejection & familial/emotional cut-off
**Knowledge &** **misconceptions about HIV** **vaccine trials**	Community consultations by national & international organizations	MSM CBO clients have more knowledge & awareness about HIV vaccines than other MSM	Confusion of VISP with actual HIV infection	Misunderstanding of placebo-controlled & double-blinding; general vaccine knowledge & attitudes
			Misconceptions aboutneed to abstain fromsex or condom usein trial	Preventive misconception
**Safety concerns & side** **effects**	VISP may cause problems for international travel & workvisas	MSM CBOs advocate forhealth & life insurancebenefitsfrom trial	VISP may introduce problems with family & partners; fear of infecting wife/partners	Fear of serious injury, disability, impotence & vaccine-induced infection
**Altruism**	Benefits to the nation	Giving back to one’s (MSM) community	Bringing respect to family	Build self-worth; martyrdom
	Combat stigma against MSM	Support MSM CBOs		

Note: CBO = community-based organization.

MSM = men who have sex with men.

VISP = vaccine-induced seropositivity.

Stigma and discrimination permeated the social ecology of WTP among MSM. Although the Delhi high court in 2008 [23] decreed that consensual sex between same-sex adults is no longer criminal, participants were concerned about prevailing negative and sometime hostile societal attitudes and institutionalized discrimination against same-sex attracted people, particularly those who are not gender-conforming [15]. Stigma operated at the community level in expressed fears of being looked down upon by one’s peers–including other MSM–as one who engages in sexual risk behaviors or is HIV-positive, and thereby one who may fuel stereotypes about MSM among the general public.

At the interpersonal level, stigma operated through fears of adverse consequences of unwanted disclosure of one’s sexuality, including family rejection (from parents, female spouses and male partners) and loss of respect and bringing shame upon one’s family. Accordingly, the stigma that permeates the social ecology of many MSM in India may effectively transform their widespread motivations based on altruism and wanting to give back to their community into liabilities.

In light of the lack of mention of family in most existing studies of WTP, the role of families in decision-making was notable. In a review of 53 HIV vaccine preparedness studies [9], only one, conducted among heterosexuals in India, noted social costs due to familial concerns [24]. In the present study, MSM described consulting with and even getting ‘permission’ from parents and partners as essential to WTP. Interestingly, MSM who were financially dependent on parents feared being cut off; and MSM whose parents were financially dependent on them feared becoming ill or injured from the trial and not being able to provide for the family. In both cases financial considerations implicate the centrality of family to WTP. MSM who did not live with their parents and were unmarried expressed reliance on the advice of community leaders and CBO staff, a type of surrogate family–and high WTP contingent on CBO endorsement.

Another pervasive concern was the safety and potential side effects of experimental HIV vaccines, as corroborated by many studies of WTP [7], [9]. Some participants understood the inherent uncertainty of clinical trials as well as the fact that a phase III trial indicated previous testing for product safety. However some MSM demanded decisive assurances that there would be *no* adverse effects. Safety concerns, however, which are generally approached as an individual-level phenomenon and might thereby be addressed by trialists through educational measures and the informed consent process, were embedded in familial concerns. Many MSM expressed WTP contingent on clinical trials providing health insurance coverage and compensation for family members in the event they are injured in a trial.

Competing considerations about monetary compensation emerged among MSM participants and community leaders. Participants expected that trial volunteers should be well compensated for their time. However, MSM CBO leaders were acutely aware of the potential for compensation that is tantamount to coercion among a client population in which most earn less than $1.50 US per day [21], half of per capita Indian income (∼$3.00 US per day) [25]. Although MSM community leaders largely supported WTP, recognizing the importance of an HIV vaccine to their communities, they were adamant that participation be truly informed and voluntary. Similar concerns about the role of financial incentives in WTP have been expressed in other low- and middle-income country sites in Africa [26], [27] and among marginalized populations in the US [28], [29].

Importantly, preventive misconception emerged across focus group participants and KIs, along with misunderstanding of key clinical trial concepts, even among those MSM previously engaged in IAVI consultations. Some participants failed to understand the meaning of “candidate” or “experimental”–that the product being tested might not be efficacious; among these, some MSM further construed clinical trials as a form of preventive intervention. Why would a trusted CBO refer them if the “intervention” was not effective? Others, while grasping the experimental nature of vaccines tested in clinical trials, failed to comprehend the meaning of placebo-controlled; they presumed that they, like everyone else, would be given the experimental vaccine, thereby enabling hope that it might work. Finally, others comprehended the meaning of experimental vaccines and placebo-controlled trials, but retained hope that they might be “lucky” and gain protection. In each of these cases the potential for increased sexual risk behaviors arose, including the ability to engage in sex work to generate income without HIV risk.

Misconceptions about HIV vaccine trials may be due in part to the fact that some key clinical trial terminology does not directly translate into Tamil or other Indian languages; and the literal or scientific translation is not comprehensible to lay participants with high school education or less who do not understand the scientific basis for randomized controlled trials (RCTs). A prominent example is “placebo”. A KI community leader who had been engaged in previous IAVI consultations noted that he did not adequately understand what “placebo” means and was unable to explain it to peer outreach staff or clients. Some participants concretely adopted the term “distilled water” as a synonym; however they went on explain that it was unfair to give some trial participants distilled water instead of the test vaccine (which, they reasoned, might be efficacious) and, moreover, unfair and unacceptable that participants would be blind to their randomization.

A second reason for misconceptions may be due to misinterpretation, or perhaps over-interpretation, of clinical trial guidelines in the absence of an understanding of the scientific (and ethical) basis for RCTs. Some MSM peer outreach workers in focus groups (who regularly interact with MSM clientele) indicated beliefs that MSM in trials should abstain from sex (as it might be made more dangerous by the experimental product), while others indicated that MSM in clinical trials should not use condoms so the efficacy of the experimental vaccine could be determined. A third possible basis for misconceptions about HIV vaccine trials is misunderstanding and folk theories about HIV transmission [30] and HIV vaccines [31]. For example, the mental model of vaccines as introducing a small dose of pathogen to train the immune system [31]–and fear that inactivated HIV may “come alive” when it comes into contact with blood–is particularly troublesome in the case of HIV.

Finally, varying perspectives emerged on WTP across different MSM subgroups. Most indicated that kothis, particularly kothis engaged in sex work, would be more likely to participate compared to double-deckers or panthis; kothis have a strong sense of community identity, are attached to MSM CBOs and often live in poverty. However, panthis and double-deckers indicated that MSM from their groups are not preoccupied by fears of disclosure of their sexuality as most are masculine-looking and perceived to be ‘heterosexual’; thus stigma may be less of a barrier to participation than among kothis. Focus group participants and KIs alike indicated that middle- and upper-class MSM, even those who self-identify as gay or bisexual, would be less likely to participate; many do not perceive themselves or their community to be at risk for HIV, they largely do not engage with MSM CBOs (generally serving lower socioeconomic MSM) and the compensation would be negligible.

### Limitations

As a qualitative study, our purpose was to explore in depth rather than to generalize; thus the findings may not apply across MSM populations in India. In particular, the sample may be more representative of lower socioeconomic MSM; middle- and upper-class MSM in India may have different perspectives on WTP. Additionally, the recruitment methods may bias the sample towards MSM who are already engaged with CBOs. MSM who are not connected with CBOs may prove harder to reach, less aware about HIV and less willing to participate. However we successfully recruited a moderately sized and diverse sample of MSM, community leaders and service providers across four languages in two large metropolitan areas in India with high HIV prevalence among MSM–feasible locales for conducting biomedical HIV prevention trials.

### Implications for Practice and Research

Implications of the social ecology of WTP among MSM in India are that trialists approach at multiple levels what might otherwise be construed largely as individual-level phenomena (e.g., side effects, safety, monetary compensation and altruism)–and thereby largely addressed with interventions targeting the individual. For example, knowledge-based approaches to address misconceptions and mitigate undue fears of side effects and safety concerns may be ineffective in supporting WTP if they do not address financial considerations regarding lost income and support for dependent family members. Societal and community stigma and familial relationships provide a crucial lens for understanding WTP among MSM in India; they also underscore the importance of confidentiality [9] and respectful relationships between trial staff and participants [32] in supporting WTP as well as retention in HIV prevention trials.

Measures at the social-structural level to reduce barriers to WTP among MSM might include mass media campaigns that aim to promote positive images of same-sex sexuality and destigmatize HIV. The Indian government has previously launched a television and billboard advertising campaign that mobilized a popular cartoon character with a play on words (in Tamil) to challenge the image of a man with many female sex partners as virile, instead designating the character as foolish and at high risk for HIV. Similar campaigns might target negative attitudes among the general public towards MSM and persons living with HIV.

At the community level, tailored interventions to reduce HIV stigma among MSM also may support WTP. Although preventive HIV vaccine trials require participants to be HIV negative, merely engaging with a trial engenders suspicions that one is HIV positive or at high risk, thereby inviting stigma within MSM communities. CBO engagement in promoting positive images of MSM who are willing to volunteer for HIV vaccine trials may help to mobilize motivations for WTP based on altruism and giving back to one’s community, as well as combat stigma [33].

At the interpersonal level, recognizing the importance of family and partner dynamics among MSM to WTP, potential volunteers might be offered voluntary opportunities to engage their male partner, either separately or as a couple, to meet with a trained trial educator or counselor to address concerns about participation. Similarly, clinical trial staff and MSM outreach workers might be trained to counsel families about their concerns if individual participants so desire.

Given the prevalence of HIV vaccine trial misconceptions and the challenges of explaining basic concepts of RCTs to individuals with low education in the context of folk beliefs about HIV, a mental models approach [31], [34] might be incorporated in future community consultation meetings with MSM in India. At the individual level, new knowledge may be more successfully integrated if it is layered onto one’s existing conceptualizations [31]. In our previous qualitative research among MSM in Chennai, for example, we identified a mental model deployed by peer educators to explain how antiretroviral medications work, using the metaphor of an egg [35]. HIV was described as entering the body’s cells and laying many eggs, akin to a mosquito with which participants are familiar. Antiretrovirals were described as preventing those eggs from hatching and baby viruses from being released. Mental models have been similarly used to support HIV prevention in Kenya, an agricultural country, invoking “zero-grazing” to signify faithfulness to one or even multiple partners within the context of monogamous or polygamous relationships [36]. It is important, however, that such mental models are founded on in depth cultural understanding and formative qualitative research conducted in situ, and deployed cautiously; they have the potential to be infused with existing folks beliefs, resulting in further misinformation rather than clarification [30], [36], [37].

Further research among MSM in India may help to prioritize the most influential factors across the social ecology of WTP as well as to explore subpopulation differences–by geography, socioeconomic status and self-identified sexuality (e.g., kothi, panthi, double-decker, gay)–to support tailored community education and recruitment efforts. Conducting recruitment solely through MSM CBOs, however, may tend primarily to reach kothis, particularly those who engage in sex work, and may be less likely to reach middle-class gay- and bisexually-identified MSM.

### Conclusion

This investigation among diverse MSM in India suggests that applying a social ecological approach to WTP may enhance the success of recruitment efforts and the ethical implementation of HIV vaccine trials. To that end, with a new HIV vaccine design program collaboration in India [3] and phase II trials under consideration, this community-based investigation supports the value of the process of trialists partnering with local CBOs that work with MSM in designing and implementing study protocols, and involving community advisory boards and other local stakeholders throughout the trial trajectory.
